# Insulin-Reactive T Cells Convert Diabetogenic Insulin-Reactive VH125 B Cells Into Tolerogenic Cells by Reducing Germinal Center T:B Cell Interactions in NOD Mice

**DOI:** 10.3389/fimmu.2020.585886

**Published:** 2020-11-12

**Authors:** James A. Pearson, Yangyang Li, Monika Majewska-Szczepanik, Junhua Guo, Li Zhang, Yu Liu, F. Susan Wong, Li Wen

**Affiliations:** ^1^ Section of Endocrinology, School of Medicine, Yale University, New Haven, CT, United States; ^2^ Diabetes Research Group, Division of Infection and Immunity, School of Medicine, Cardiff University, Cardiff, United Kingdom; ^3^ Department of Endocrinology, Sir Run Run Shaw Hospital, Nanjing Medical University, Nanjing, China; ^4^ Department of Medical Biology, Jagiellonian University Medical College, Krakow, Poland; ^5^ Department of Rheumatology, People's Liberation Army (PLA) General Hospital, Beijing, China

**Keywords:** insulin, Type 1 diabetes, B cells, T cells, TGFβ, germinal center

## Abstract

Insulin is a key autoantigen in Type 1 Diabetes (T1D), targeted by both T and B cells. Therefore, understanding insulin-specific T:B cell interactions is important. We have previously reported an insulin-reactive CD4+ T cell, (designated 2H6). Unlike other insulin-reactive T cells, 2H6 cells protect non-obese diabetic (NOD) mice from T1D development, mediated by TGFβ. To investigate insulin-specific T:B cell interactions, we bred 2H6αβ T cell receptor transgenic NOD mice (2H6) with the insulin-reactive B cell receptor transgenic NOD mice (VH125), generating 2H6VH125 NOD mice. Similar to 2H6 mice, 2H6VH125 mice are protected from T1D development. Interestingly, VH125 B cells did not alter the phenotype of 2H6 T cells; however, 2H6 T cells significantly altered the VH125 B cells by reducing the insulin-reactive non-germinal center (GC) and GC B cells, as well as MHC and costimulatory molecule expression on the B cells. Furthermore, the B cells in 2H6VH125 NOD mice exhibited increased non-insulin-specific and a class switched IgG isotype, which can be recapitulated *in vivo* in Rag-deficient NOD mice by adoptive transfer. *In vitro*, VH125 B cells from 2H6VH125 mice suppressed the proliferation of 2H6 T cells to insulin antigen but enhanced TGFβ secretion by 2H6 T cells from 2H6VH125 mice compared to 2H6 mice. In summary, our data showed that 2H6 CD4+ T cells alter the phenotype and function of insulin-reactive B cells from pathogenic to tolerogenic cells. In turn, VH125 B cells also modulate the function of the 2H6 T cells. Thus, promoting the interactions between antigen-specific regulatory T cells and B cells may lead to protection from T1D.

## Introduction

Insulin is known to be a major autoantigen contributing to the development of Type 1 diabetes (T1D) in both humans (1–5) and Non-obese diabetic (NOD) mice (6–10). This autoantigen is recognized by CD4+ (6, 8) and CD8+ (3, 9) T cells, and B cells (11, 12). Furthermore, the second most important gene determining susceptibility to T1D in humans (IDDM2) influences the level of proinsulin expression in the thymus (13, 14). Graded deficiency in insulin gene copy numbers leads to a higher proportion of insulin-reactive T cells escaping thymic negative selection in C57BL/6 mice (15) and these cells cause accelerated diabetes in NOD mice (10).

Several insulin-reactive T cell clones are pathogenic (3, 6, 9, 16–18), whereas the 2H6 T cell clone, isolated from the islets of NOD mice is regulatory and protects mice from T1D development, mediated by TGFβ (8, 19). 2H6 recognizes overlapping Insulin B chain peptides 12–25 and 9–23, a core sequence shared by insulin-reactive pathogenic T cell clones (7, 9). Interestingly, mutating the tyrosine to alanine at position 16 of the insulin B chain prevented antigen recognition by pathogenic insulin-reactive CD4+ and CD8+ T cells and protected NOD mice from developing diabetes (10, 20, 21), demonstrating the importance of this B chain region of insulin in T1D development.

Anti-insulin autoantibodies have been an important biomarker for prediction and diagnosis of T1D development in humans (22–25). Insulin-reactive B cells in NOD mice can also alter the development of T1D (12). B cell receptor (BCR) transgenic mice with a fixed IgM heavy chain, VH125, known to bind to insulin, increased the population of insulin-specific B cells in NOD mice but not in non-autoimmune prone C57BL/6 mice (11, 12). To address the role of class-switching on VH125 B cells, Williams and co-authors generated C57BL/6 mice expressing VDJH-125 site-directed to the native IgH locus (VH125^SD^) (26). The B cells in these mice exhibited impaired proliferation to insulin and failed to produce IgG antibodies upon insulin immunization; however, this could be overcome when both BCR and toll-like receptors (TLRs) were stimulated simultaneously. Using a double transgenic Rag^-/-^NOD mouse model that expresses the T cell receptor (TCR) of the pathogenic insulin B12-20-reactive CD4+ T cell (8F10) and the VH125 BCR, Wan and colleagues demonstrated that the presence of insulin- reactive pathogenic CD4 T cells enables the class switching (VH125^SD^) of VH125 B cells (27). Using this model, the authors also demonstrated insulin-specific T:B cell interactions (27). 8F10 VH125^SD^ mice have increased anti-insulin germinal center B cells and can undergo IgG class switching when both the T cells and B cells are antigen-restricted. However, the role of regulatory insulin-specific T cells in modulating insulin-reactive VH125 B cells is still unknown. To fill this knowledge gap, we generated 2H6VH125 NOD mice encompassing the 2H6 TCR and heavy chain of 125 BCR transgenes.

Our study revealed that the presence of 2H6 T cells not only reduced the proportion of insulin-reactive B cells, but also reduced insulin binding to the B cells. Moreover, the presence of 2H6 T cells decreased the ability of B cells to present the antigen to insulin-reactive T cells. Interestingly, in the presence of VH125 B cells, 2H6 T cells had significantly enhanced TGFβ secretion but reduced proliferation in response to antigen stimulation *in vitro* compared to the 2H6 T cells that developed in the absence of VH125 B cells. Furthermore, the presence of both insulin-reactive TCR and BCR lowered the proportion of germinal center (GC) B cells and the expression of PDL1-PD1 molecules, particularly in the pancreatic lymph nodes (PLNs). We also found that some of the VH125 B cells in the 2H6VH125 NOD mice underwent IgG class switching, unlike VH125 B cells from VH125 NOD mice. Our data suggest that 2H6 T cells regulate their interactions with the insulin-specific B cells and alter the pathogenic VH125 B cells to favor TGFβ production by 2H6 T cells, and thus, mediate diabetes protection. Therefore, modulating antigen-specific T:B cell interactions may be of benefit in optimizing antigen-specific therapies, to promote protection and minimize autoimmunity.

## Materials and Methods

### Mice

NOD/Caj mice were originally obtained from the Jackson Laboratory and have been maintained at Yale University for over 30 yrs. 2H6TCRαβ NOD mice (designated 2H6; expressing the TCRα chain *Vα6/Jα45/Cα* and the TCRβ chain *Vβ14/Dβ1.1/Jβ2.3/C*β*2*) were generated, as previously described (8, 19), and maintained at Yale University. VH125 NOD mice, expressing the heavy chain of the insulin-reactive monoclonal antibody (mAb) 125 were generated by Dr James Thomas (Vanderbilt University) (12) and obtained from the Jackson Laboratory (Bar Harbor, Maine). Approximately >95% of these VH125 B cells were IgM^a^, indicating a high level of allelic exclusion (data not shown). 2H6VH125 NOD mice were obtained by breeding 2H6 NOD mice with VH125 NOD mice. Recombinase-activating-gene deficient NOD (Rag^-/-^NOD) mice were originally obtained from the Jackson Laboratory and maintained at Yale University. All the mice used in this study were kept in specific pathogen–free (SPF) conditions, in a 12-hour dark/light cycle and housed in individually-ventilated filter cages with free access to water and autoclaved food at the animal facility of Yale University. The Yale University Institutional Animal Care and Use Committee approved the usage of animals and the procedures used in this study.

### Diabetes Monitoring

Mice were monitored for glycosuria, from 12-weeks of age, for spontaneous diabetes development, with diabetes confirmed with a blood glucose >250 mg/dl.

### Histology

Pancreata from 12-week-old female mice were collected and formalin-fixed. Sections from 2–3 different layers within each pancreata were cut and haemotoxylin and eosin stained. Islets were scored under light microscopy by a blinded scorer.

### Cell Staining and Flow Cytometry

Single cell suspensions were prepared from splenocytes and pancreatic lymph nodes (PLNs) harvested from 8-week-old mice. Cells were incubated with Fc block (2.4G2) for 10 min at room temperature prior to mAb staining and a cell viability dye (Zombie Aqua Fixable viability dye, BioLegend) for 30 min at 4°C. All the antibodies used in this study were from Biolegend and diluted 1:400 to 1:500 unless otherwise stated. T cells were stained with mAbs to CD4 (GK1.5), CD25 (PC61), CD69 (H1.2F3), PD1 (29F.1A12), CD8α (53-6.7), CD40L (MR1), CXCR5 (L138D7), CCR7 (4B12), and TCR Vβ14 (14-2; BD Biosciences). B cells were stained with mAbs to CD19 (6D5), B220 (RA3-6B2), MHCII (10-3.6), CD40 (3/23), CD21 (7E9), CD23 (B3B4), GL7 (GL7), CD80 (16-10A1), CD86 (GL-1), PDL-1 (10F.9G2), and PNA (Sigma). Insulin-FITC (Sigma) was used for the detection of insulin-reactive B cells (at a dilution of 1:2000) as previously described (28). Cells were then washed (with an additional wash if stained with insulin-FITC), resuspended and examined on a BD LSRII flow cytometer on the same day. Fluorescence minus one (FMO) controls were used in the initial compensation and all the cells were gated on single live cells. Data were analyzed with Flowjo version 8.8.6 (Treestar).

### Insulin Auto-Antibody Measurements by Sandwich ELISA

Peripheral blood from 8-week-old donor mice was collected and kept at 4°C for 4 h to clot, prior to serum separation by centrifugation (8000rpm, 5 min, room temperature). 96-well plates were coated with 4 μg/ml insulin (Humulin N, Lilly) overnight at 4°C, followed by washing with PBS (0.05% TWEEN). The plates were then blocked with PBS containing 1% BSA, for 1 hour at room temperature. After an additional wash, diluted sera (1:100) were added to the plates and incubated for 2 h at room temperature. Post-wash, alkaline phosphatase (AP)-conjugated Ig, IgG or IgM (1:1000, Southern Biotech) was added for a further 2 h incubation at room temperature. After washing, PNPP substrate (Sigma) was added. The enzymatic reaction was stopped using 1M NaOH. Plates were read at OD405 using a microplate spectrophotometer (Perkin Elmer) and data were plotted, based on the optical density (OD) values.

### Measurement of Total Immunoglobulins (Ig) and Different Isotypes by Direct ELISA

Serum samples were prepared and diluted as described above and used to coat the plate overnight at 4°C. Known concentrations of Ig or isotypes were also coated for standard curves. The plates were washed and blocked the next day, prior to the addition of pre-titrated Ig-AP, IgG-AP or IgM-AP. The ELISA was completed by enzymatic reaction using PNPP substrate (see above). The plates were read at OD405 using a microplate spectrophotometer (Perkin Elmer) and sample concentrations were determined using standard curves for each isotype.

### Cytokine Measurements by ELISA

Cell culture supernatants and/or sera were assessed for TGFβ1 (Mouse TGFβ1 DuoSet ELISA, R&D Systems) and IL-21 (Mouse IL-21 ELISA Ready-SET-GO! Kit, eBioscience) following the manufacturers’ protocols.

### Cell Isolation

B cells and CD4+ T cells were isolated from the spleen of 8-week-old donor mice. B cells were purified by negative selection using the EasySep Mouse B cell Isolation kit (StemCell Technologies). Splenic CD4+ T cells were purified using hybridoma supernatants containing mAbs to CD8 (TB105) and I-A^g7^ (10.2.16, recognizing MHC class II I-A of NOD mouse), generously provided by the late Charles Janeway Jr. (Yale University) for 30 min at 4°C. Cells were then washed in PBS and incubated for 45 min on ice with magnetic beads conjugated with goat anti-mouse IgG, goat anti-mouse IgM, or goat anti-rat IgG (QIAGEN). CD4+ T cells were magnetically isolated using negative selection. The purity for all cells was 95–99% as verified by flow cytometry.

### qPCR

Total RNA was extracted from purified splenic B cells (see above) of 8-week-old donor mice using the RNeasy Mini Kit (Qiagen), prior to cDNA generation with the iScript^TM^ cDNA synthesis kit (Bio-Rad). The transcriptional levels of genes of interest were detected by qPCR on an iCycler (Bio-Rad). qPCR data were presented as fold change using the 2**-**ΔΔCt method and normalized with the GAPDH values. Primer sequences: *aicda* (F- 5’CCTAAGACTTTGAGGGAGTCAA3’, R- 5’CACGTAGCAGAGGTAGGTCTC3’), *tgfbetar1* (F- 5’TCACATTGTGCCAATGGAAT3’, R- 5’AAAATTGCAAACCTGGTGGA3’), *il6* (F- 5’CCAGAGATACAAAGAAATGATGG3’, R- 5’ACTCCAGAAGACCAGAGGAAAT3’), and *gapdh* (F*-* 5’AGGTCGGTGTGAACGGATTTG3’, R-5’GGGGTCGTTGATGGCAACA3’).

### Cell Proliferation

Purified CD4+ T cells and B cells were co-cultured at 1:1 ratio, in the presence of insulin B chain 12–25 peptide or denatured insulin (heat inactivated at 95°C for 5 minutes) for 48 h. Culture supernatants were collected prior to the addition of ^3^H-thymidine and the cells were cultured for a further 18 h. Cell proliferation was assessed by ^3^H-thymidine incorporation, quantified by a β counter. Data were normalized as corrected CPM whereby the background (cultures without antigen) was subtracted.

### Adoptive Transfer

Four-week-old Rag-/-NOD mice were infused (*i.v.*) with 3x10^6^ purified CD4+ T cells, together with the same number of purified B cells (1:1 ratio). Recipient mice were harvested for experiments 4 weeks post-transfer.

### Statistics

Multiple Student’s t tests with FDR correction were used to determine the statistical significance for all the results presented, except **Supplementary Figure 1** where a Log-rank (Mantel-Cox) test and chi-square test was used and **Figure 4A** where two-way ANOVA was used. Statistical analysis was conducted using Prism 8.

## Results

### The Presence of Insulin-Reactive VH125 BCR Does Not Affect the Phenotype of Insulin-Reactive 2H6 T Cells

**Figure 1:**
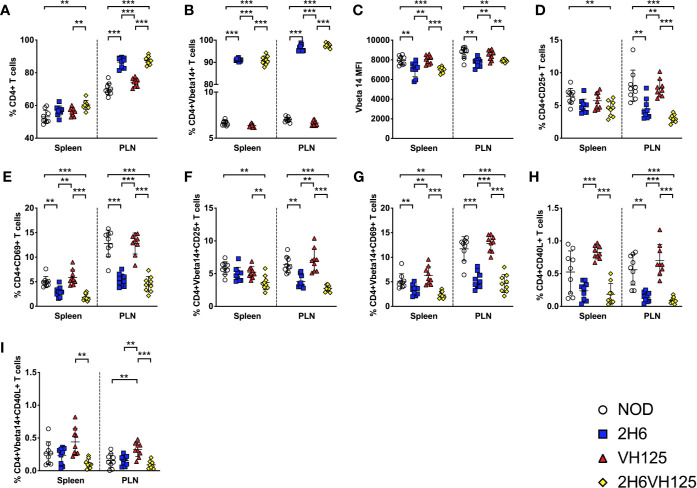
VH125 BCR transgene does not influence 2H6 T cell phenotype. Splenocytes and cells from the pancreas draining lymph nodes (PLN) were harvested from 8-week-old mice and stained using antibodies for flow cytometry. **(A)** % of live single CD4+CD8-CD19- T cells. **(B, C)** % and mean fluorescent intensity (MFI) of TCRVβ14+ (the TCRVβ chain used in the 2H6 TCR transgene) in CD4+ T cells gated from **(A)**. **(D–G)** % of CD4+CD25+ T cells and CD4+CD69+ T cells gated on total CD4+ T cells and CD4+ TCRVβ14+. (**H–I**; % of CD4+CD40L+ T cells gated on total CD4+ T cells and CD4+ TCRVβ14+. Data were generated from 9 to 10 individual mice pooled from 4 to 5 independent experiments. Data from left to right: NOD, 2H6, VH125, and 2H6VH125 are shown in this order in each plot. Data from NOD mice are shown as a non-transgenic mouse comparison. Data were assessed for significance using multiple T tests and FDR correction. **P < 0.001, ***P < 0.0001.

To investigate the interaction of insulin-reactive regulatory T cells with B cells, we bred 2H6αβ TCR transgenic NOD mice (2H6) with VH125 BCR transgenic mice (VH125) to generate 2H6αβVH125 transgenic NOD mice (2H6VH125). 2H6 NOD mice are protected from T1D development (19) whereas VH125 NOD mice have accelerated T1D development (12). We hypothesized that 2H6VH125 mice would have a reduced incidence of diabetes. To test our hypothesis, we monitored incidence of diabetes in these mice. Interestingly, 2H6VH125 mice were completely protected from diabetes development, similar to 2H6 NOD mice (**Supplementary Figure 1A**). Furthermore, like 2H6 mice, there was minimal evidence of insulitis in the 2H6VH125 NOD mice (**Supplementary Figure 1B**). To determine the impact of the VH125 B cells on the T cell compartment in the 2H6VH125 mice, we examined the phenotype of T cells in different lymphoid tissues. We found that 2H6VH125 NOD mice had reduced splenic cellularity, although there were no differences in cellularity of the PLNs compared to both the parental TCR and BCR transgenic NOD mice (**Supplementary Figure 2A**). Mesenteric lymph nodes and Peyer’s Patches were also harvested and found to have similar trends (data not shown). The expression of the 2H6 TCR transgene, with or without the co-expression of the VH125 BCR, led to significant increases in the proportion of CD4+ T cells in the PLN compared to non-transgenic NOD mice and VH125 NOD mice (**Figure 1A**). As expected, in the parental 2H6 TCR transgenic mice, the majority of the T cells were 2H6 transgenic TCRVβ14+, the surrogate marker for clonotypic 2H6 TCR, and the expression of VH125 BCR had no effect on the selection of these TCRVβ14+ T cells (**Figure 1B**). However, the intensity of TCR expression, presented as mean fluorescence intensity (MFI), was reduced in transgenic 2H6 T cells compared to the non-transgenic TCRVβ14+ cells (**Figure 1C**), which comprised ~3–7% of total peripheral CD4+ T cells in wild-type polyclonal NOD [**Figure 1B** and (19)] and VH125 mice (**Figure 1B**). Furthermore, the expression of the 2H6 TCR transgene reduced the T cell activation, particularly in the PLNs, as demonstrated by the reduced proportion of CD25 and CD69 in both the total CD4+ T cells (**Figures 1D, E**) and the TCRVβ14+ CD4 T cell population (**Figures 1F, G**) compared to wild-type NOD and VH125 mice, which express polyclonal TCRs. Finally, we analyzed the expression of CD40L, an important ligand for T:B cell interactions and B cell maturation, and found reduced CD40L on the CD4+ T cells in the 2H6-expressing mice compared to NOD and VH125 mice (**Figures 1H–I**). Interestingly, CD40L expression on CD4+TCRVβ14+ T cells in VH125 NOD mice, but not 2H6VH125 NOD mice, was increased, suggesting that non-2H6-restricted TCRVβ14-expressing CD4+ T cells may provide more costimulation to the B cells in VH125 NOD mice (**Figure 1I**). However, the insulin-reactive BCR does not appear to promote the activation of the insulin-reactive 2H6 T cells in the 2H6VH125 NOD mice, which may contribute to the disease-protected phenotype.

### Insulin-Reactive 2H6 TCR Inhibits the Insulin-Reactive B Cell Populations and Alters B Cell Phenotype

**Figure 2:**
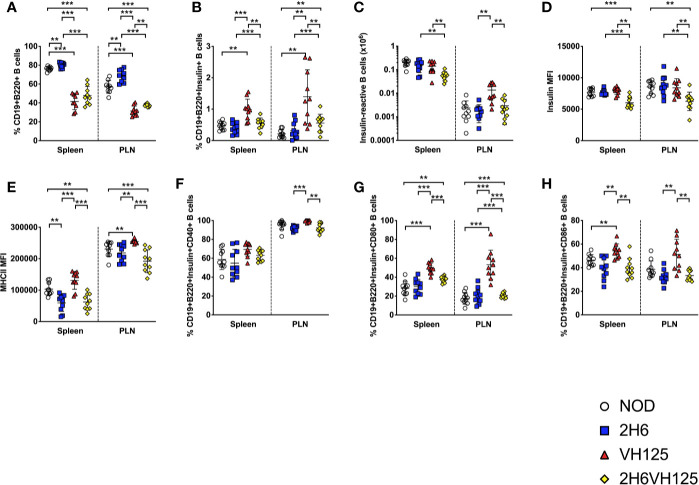
The 2H6 TCR transgene strongly reduces frequency of insulin-reactive VH125 B cells and their costimulatory molecule expression. Splenocytes and cells from the pancreas draining lymph nodes (PLN) were harvested from 8-week-old mice and stained using antibodies for flow cytometry. **(A)** % of live single CD19+B220+TCRβ- B cells. **(B)** % of insulin-reactive B cells identified by binding to insulin-FITC in CD19+B220+ B cells gated from **(A)**. **(C)** Absolute number of Insulin-reactive B cells. **(D, E)** Mean fluorescent intensity (MFI) of insulin-FITC and MHCII (IA^g7^), gated within the insulin-reactive B cells in **(B)**. **(F–H)** % of CD40, CD80, and CD86 gated within the insulin-reactive B cells. Data were generated from 10 individual mice pooled from 4 to 5 independent experiments. Data from left to right: NOD, 2H6, VH125, and 2H6VH125 are shown in this order in each plot. Data from NOD mice are shown as a non-transgenic mouse comparison. Data were assessed for significance using multiple T tests and FDR correction. **P < 0.001, ***P < 0.0001.

Although the presence of the insulin-reactive B cells did not appear to have substantial effects on the 2H6 cells, we asked reciprocally whether the changes in T cells, as a result of TCR transgene expression, affected B cell development. This was particularly related to the finding of the reduced proportion of CD40L+ T cells in 2H6 and 2H6VH125 NOD mice. To test our hypothesis, we examined the B cell compartment in the four strains of NOD mice. Interestingly, we found that both VH125 and 2H6VH125 NOD mice had reduced CD19+B220+ B cells compared to NOD and 2H6 NOD mice (**Figure 2A**). Next, we studied the impact of the 2H6 TCR on the insulin-specific B cells. We demonstrated that the 2H6 TCR significantly reduced the insulin-reactive B cells in 2H6VH125 NOD mice compared to VH125 BCR transgenic mice, even though the proportion and number of these insulin-reactive B cells was still higher than in NOD and 2H6 NOD mice (**Figures 2B, C** and **Supplementary Figures 2B, C**). However, the VH125 BCR expression reduced the follicular B cells and enhanced the proportion of marginal zone B cells in both VH125 BCR and 2H6VH125 transgenic mice (**Supplementary Figures 2D, E**), supporting an earlier report (29). Further investigation of the insulin-reactive B cells revealed reduced insulin binding to the insulin-reactive B cells in 2H6VH125 NOD mice compared with VH125 transgenic mice (**Figure 2D**). The presence of 2H6 TCR also led to the reduced expression of MHCII on the surface of insulin-reactive B cells isolated from both 2H6 and 2H6VH125 NOD mice (**Figure 2E**). MHCII expression was also significantly reduced on the total B cell population in 2H6VH125 NOD mice (**Supplementary Figure 2F**). Moreover, the presence of the 2H6 transgene reduced the expression of costimulatory molecules (CD40, CD80, CD86) on the insulin-reactive B cells compared to VH125 NOD mice (**Figures 2F–H**). This effect was also noted on the total B cell population (**Supplementary Figures 2G–I**). Therefore, the presence of the 2H6 T cell transgene has profound effects on both the T cell and B cell phenotype, promoting a more tolerogenic environment *in vivo*.

### Insulin-Reactive T Cells Induce the Insulin-Reactive B Cells to Undergo Non-Insulin-Specific Class Switching

**Figure 3:**
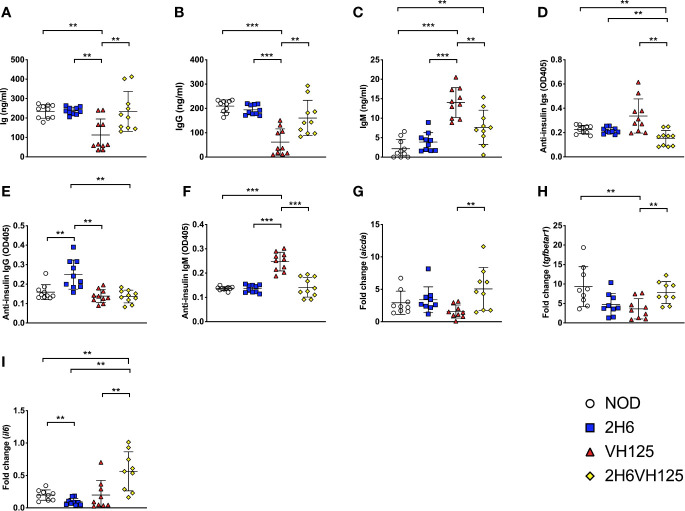
2H6 TCR transgene promotes IgG class switching in VH125 B cells. **(A–F)** Sera from 8-week-old mice were tested for total Ig **(A)**, IgG **(B)**, IgM **(C)**, and insulin-specific autoantibodies of the same isotypes (D to F). RNA from splenic B cells was isolated and cDNA synthesized, prior to performing qPCR, to quantify the *aicda* **(G)**, *tgfbeta1* **(H)**, and *il6* **(I)** genes. qPCR data are presented as fold change using the 2**-**ΔΔCt method and normalized using the GAPDH values. Data were generated from 9 to 10 individual mice pooled from two independent experiments. Data from left to right: NOD, 2H6, VH125, and 2H6VH125 are shown in this order in each plot. Data from NOD mice are shown as a non-transgenic mouse comparison. Data were assessed for significance using multiple T tests and FDR correction. **P < 0.001, ***P < 0.0001.

Next, we determined whether expression of the insulin-reactive TCR affects the B cell secretion of polyclonal immunoglobulins (Igs). Expression of the 2H6 TCR alone did not affect the levels of total Igs or different isotypes of Ig compared with wild-type NOD mice (**Figures 3A–C**). However, expression of the VH125 BCR significantly reduced total circulating Ig (**Figure 3A**), and IgG (**Figure 3B**) compared with wild type NOD and 2H6 TCR transgenic mice. In contrast, expression of VH125 significantly enhanced IgM production (**Figure 3C**), which was expected, as VH125 BCR encodes an IgM isotype antibody. However, it is interesting that co-expression of 2H6 TCR and VH125 BCR altered the ability of some B cells to secrete other isotypes of Ig including IgG, (**Figures 3A, B** and **Supplementary Figures 3A–C**) but suppressed IgM production by VH125 B cells (**Figure 3C**). Interestingly, serum IgA was significantly increased in all the transgenic mice compared to the non-transgenic NOD mice (**Supplementary Figure 3D**). As the expression of insulin-reactive TCR modified the polyclonal antibody production by VH125 B cells, we hypothesized that insulin-specific antibodies would also be affected. To test this, we assessed anti-insulin antibodies in the serum of the NOD mice, with and without insulin-reactive TCR and/or BCR. We found that 2H6VH125 NOD mice had the lowest concentration of total anti-insulin-Ig (**Figure 3D**), which is in sharp contrast to the total circulating Igs (**Figure 3A**). Interestingly, while the 2H6 TCR alone promoted insulin-specific IgG production by polyclonal B cells compared to all other mice (**Figure 3E**), expression of VH125 alone resulted in increased insulin-specific IgM production, as anticipated (**Figure 3F**). Further investigation into IgG subclasses revealed that the 2H6VH125 mice had the most decreased insulin-specific IgG1, while insulin-specific IgG2a and IgG2b were increased compared to VH125 mice (**Supplementary Figures 3E–G**). However, 2H6 mice had the highest concentration of anti-insulin IgG antibodies (**Figure 3E**), of IgG2a and IgG2b isotypes (**Supplementary Figures 3F–G**). The presence of the 2H6 transgene only, in the T cells, which recognize insulin, increased insulin-specific B cell autoantibodies, potentially due to the increased frequency of insulin-reactive T cells; however, despite this, the T cell-mediated protection is dominant. This may suggest that anti-insulin autoantibodies can be induced by both pathogenic and regulatory mechanisms. We also observed increased anti-insulin IgA in all the transgenic strains compared to the wild type NOD (**Supplementary Figure 3H**). To verify the observed class switching in B cells from 2H6VH125 NOD mice, we evaluated the gene expression of *aicda*, encoding an enzyme required for mediating class switching (30), as well as genes related to cytokines involved in class switching by qPCR. We found that B cells from 2H6VH125 mice had increased expression of *aicda, tgfbetar1* and *il6* compared to B cells from VH125 NOD (**Figures 3G–I**), demonstrating the potential for enhanced class switching. Our data also suggest the altered cytokine milieu may promote IgM to IgG class switching in the 2H6VH125 NOD mice.

### 2H6 TCR Reduces Antigen Presentation Function of B Cells While Enhancing TGFβ Secretion

**Figure 4:**
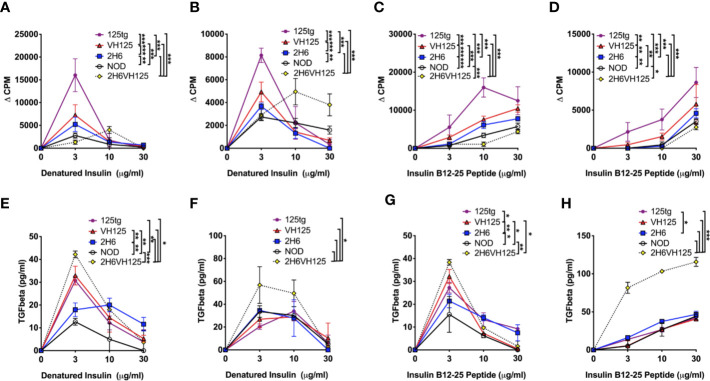
2H6 TCR transgene strongly reduces antigen presentation by insulin-reactive VH125 B cells and increases anti-inflammatory TGFβ secretion. Splenic B cells were negatively isolated and cultured with bead-purified CD4+ T cells from 8-week-old mice in a 1:1 ratio. Cells were co-cultured with denatured insulin or insulin B chain 12-25 peptide at various concentrations for 48 h prior to supernatant collection and ^3^H-thymidine addition. Proliferation was measured by ^3^H-thymidine incorporation and plotted as ΔCPM, where the background CPM (cells without antigen) was subtracted from the CPM of cells with antigen. **(A, B)** 2H6 CD4+ T cells isolated from 2H6 NOD mice **(A)** or 2H6 T cells isolated from 2H6VH125 NOD mice **(B)** co-cultured with B cells from various donors and denatured insulin. **(C, D)** 2H6 CD4+ T cells isolated from 2H6 NOD mice **(C)** or 2H6 T cells isolated from 2H6VH125 NOD mice **(D)** co-cultured with B cells from various donors and insulin B chain 12–25 peptide. **(E–H)** TGFβ measurements from the supernatants of the respective cultures in **(A–D)**. Data were generated from three independent experiments (n = 2–3 mice/experiment). Data from NOD mice are shown as a non-transgenic mouse comparison. Data were assessed for significance using two-way ANOVA. *P < 0.05, **P < 0.01, ***P < 0.0001.

Having identified that insulin-reactive 2H6 T cells suppressed insulin-reactive B cell production of anti-insulin antibodies, we asked if 2H6 T cells also modulate the antigen presentation function of insulin-reactive B cells. To answer this question, we investigated 2H6 T cell responses to insulin antigen presented by B cells from i) 125 transgenic (125tg) NOD mice, which express both light and heavy chains of 125 BCR; ii) VH125 NOD mice, which express only the heavy chain of 125 BCR and iii) 2H6VH125 NOD mice, which express only the heavy chain of 125 BCR and the 2H6 TCR, iv) 2H6 NOD mice, which have polyclonal BCR and v) wild type NOD mice, which have polyclonal BCR. To prevent possible bioactivity of insulin on the cultured cells, we used heat-denatured insulin. We found that 2H6 CD4+ T cells responded best to the denatured insulin antigen presented by the 125 B cells. This was followed by VH125 B cells. Interestingly, B cells from 2H6VH125 NOD mice had the least capacity to induce proliferation of 2H6 T cells at the antigen concentration that induced a higher response by other B cells (3 μg/ml, **Figure 4A**), although this improved at a higher insulin concentration (10 μg/ml, **Figure 4A**). Similar patterns were also seen when 2H6 CD4+ T cells from 2H6VH125 mice were cultured with B cells from the 5 different mouse strains in the presence of denatured insulin protein (**Figure 4B**). Furthermore, 2H6 T cells from 2H6 NOD mice appeared to have a greater proliferative response to antigen (higher Δ CPM) compared with the 2H6 cells from 2H6VH125 mice. This was particularly evident when B cells from 125 mice were used as antigen presenting cells (APCs) (**Figure 4A** compared with **Figure 4B**). To further confirm the antigen specificity, we examined the response of 2H6 T cells from 2H6 or 2H6VH125 mice to insulin B chain 12–25 peptide presented by B cells isolated from the 5 different NOD mouse strains. Similar to the proliferative response to denatured insulin protein, the CD4+ T cells from 2H6 NOD mice proliferated more strongly to the peptide (**Figure 4C**, higher Δ CPM) compared with the 2H6 CD4+ T cells from 2H6VH125 NOD mice (**Figure 4D**) when B cells from 125 NOD mice were used as APCs. Again, the B cells from 2H6VH125 NOD mice showed the lowest ability to induce proliferation of 2H6 CD4+ T cells (**Figures 4C, D**). Given that diabetes protection by 2H6 T cells is mediated through TGFβ (19), we hypothesized that the weaker T cell proliferative response could be due to a stronger TGFβ-induced immune response. Indeed, we found that TGFβ production in the supernatants of the above cell cultures was highest in the CD4+ T cell culture supernatants when 2H6VH125 NOD mice were responders (**Figures 4E–H**). Furthermore, the highest levels of TGFβ secretion from the 2H6VH125 cell cultures in response to insulin peptide occurred at the antigen concentration that induced lower CD4+ T cell proliferation compared to the CD4+ T cells from the other mouse strains. This may suggest that the low proliferative response of CD4+ T cells from 2H6VH125 NOD mice is likely to be a result of high TGFβ production.

### 2H6 TCR Alters Germinal Center T:B Interactions

**Figure 5:**
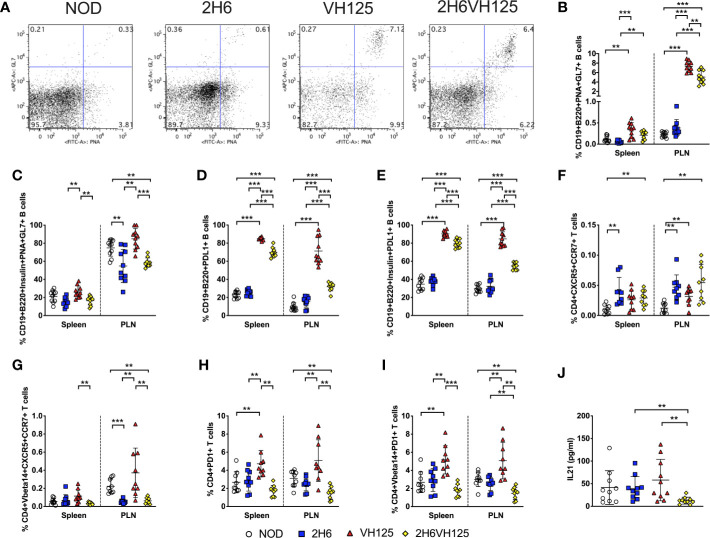
2H6 TCR transgene alters germinal center interactions in vivo. Splenocytes and cells from the pancreas draining lymph nodes (PLN) were harvested from 8-week-old mice and stained using antibodies for flow cytometry. **(A)** Representative flow plots showing PNA vs. GL7 staining of CD19+B220+ B cells, gated from live single TCRβ- cells with summary **(B)**. **(C)** Insulin-reactive germinal center B cells % [gated as **(B)** on Insulin+CD19+B220+ B cells]. **(D)** % CD19+B220+PDL1+ B cells gated as in **(B)** and insulin-reactive CD19+B220+PDL1+ B cells **(E)**. **(F, G)** % of live single CD4+CD8-CD19-CXCR5+CCR7+ T cells gated on total CD4+ T cells **(F)** and TCRVbeta14+ CD4+ T cells **(G)**. **(H, I)** % of live single CD4+CD8-CD19-PD1+ T cells gated on total CD4+ T cells **(H)** and TCRVbeta14+ CD4+ T cells **(I)**. IL21 was measured in sera from 8-week-old mice by ELISA **(J)**. Data were generated from 9 to 10 individual mice pooled from 4 to 5 independent experiments. Data from left to right: NOD, 2H6, VH125, and 2H6VH125 are shown in this order in each plot. Data from NOD mice are shown as a non-transgenic mouse comparison. Data were assessed for significance using multiple T tests and FDR correction. **P < 0.001, ***P < 0.0001.

We have shown that 2H6 TCR promoted class switching of polyclonal B cells but inhibited anti-insulin antibody production and antigen presentation by insulin-reactive B cells (**Figure 3** and **Supplementary Figure 2**). These results provide strong evidence that insulin-reactive T cells interact with insulin-reactive B cells. We, therefore, hypothesized that the germinal center (GC) reaction, which shapes the development of specialized GC B cell and follicular helper T cell (Tfh) immune responses *in vivo* (31, 32), is also affected. To test our hypothesis, we first examined GC B cells, characterized by PNA and GL7 dual expression, in the spleen and PLN. We found that the expression of VH125 sharply increased the proportion of total GC B cells in VH125 and 2H6VH125 mice compared to NOD and 2H6 NOD mice, which do not express insulin-reactive BCR transgenes; however, the co-presence of the 2H6 TCR in 2H6VH125 mice led to a lesser increase of the GC B cells (**Figures 5A, B**). Furthermore, assessment of the insulin-reactive B cells revealed that this lesser increase seen, induced in the 2H6VH125 mice, was also clearly seen in the insulin-specific GC B cells, especially in PLN (**Figure 5C**). As PDL1 on B cells, is an important molecule that regulates Tfh-mediated autoimmunity (33), we examined expression of PDL1 on the B cells of the different mouse strains. Expression of 2H6 alone did not have an obvious effect on PDL1 expression on B cells, whereas the presence of VH125 markedly increased PDL1-expression on B cells (**Figure 5D**). Again, the presence of 2H6 TCR suppressed PDL1 expression in total and insulin-specific B cells in 2H6VH125 mice compared with these B cells in VH125 mice (**Figures 5D, E**). However, the percentage of PDL1+ B cells was still significantly higher in 2H6VH125 NOD mice compared with the NOD and 2H6 NOD mice (**Figures 5D, E**). Next, we investigated the Tfh cells, characterized by expression of CCR7 and CXCR5, in the 4 NOD mouse strains. We found that 2H6-expressing mice had more Tfh cells than NOD mice (**Figure 5F**), suggesting the increase was related to the 2H6 TCR. Both 2H6 and 2H6VH125 mice had increased Tfh cells compared to VH125 mice, when assessed by a two-tailed T test, but this did not reach significance after multiple T test and FDR corrections were applied. Interestingly, when examining TCRVβ14-specific Tfh cells, we found that NOD and VH125 mice, which do not express 2H6 TCR, had a higher number of TCRVβ14+ Tfh cells, especially in the PLN (**Figure 5G**). Next, we investigated the expression of PD1, the receptor for PDL1, on total CD4+ T cells and TCRVβ14+ T cells. It is interesting that expression of the VH125 BCR increased the number of PD1+ total and TCRVβ14+ CD4+ T cells (**Figures 5H, I**) in VH125 mice, whereas expression of the 2H6 TCR reduced PD1 expression, especially in 2H6VH125 NOD mice (**Figures 5H, I**). IL-21, produced by Tfh cells (34), is an important cytokine for GC formation (35), and we found that 2H6VH125 NOD mice had the lowest level of circulating IL-21 among the 4 mouse strains (**Figure 5J**), which is in line with the lowest proportion of Tfh cells (**Figures 5G–I**). Thus, the reduction of GC B cells in 2H6VH125 NOD mice is potentially due to the reduced IL-21, in addition to reduced interactions between the Tfh cells and GC B cells via the PD1 and PDL-1 pathway.

#### Co-Transfer of 2H6 T Cells and VH125 B Cells to Rag-Deficient NOD Mice Recapitulates the Cellular Phenotypes of 2H6VH125 NOD Mice In Vivo

**Figure 6:**
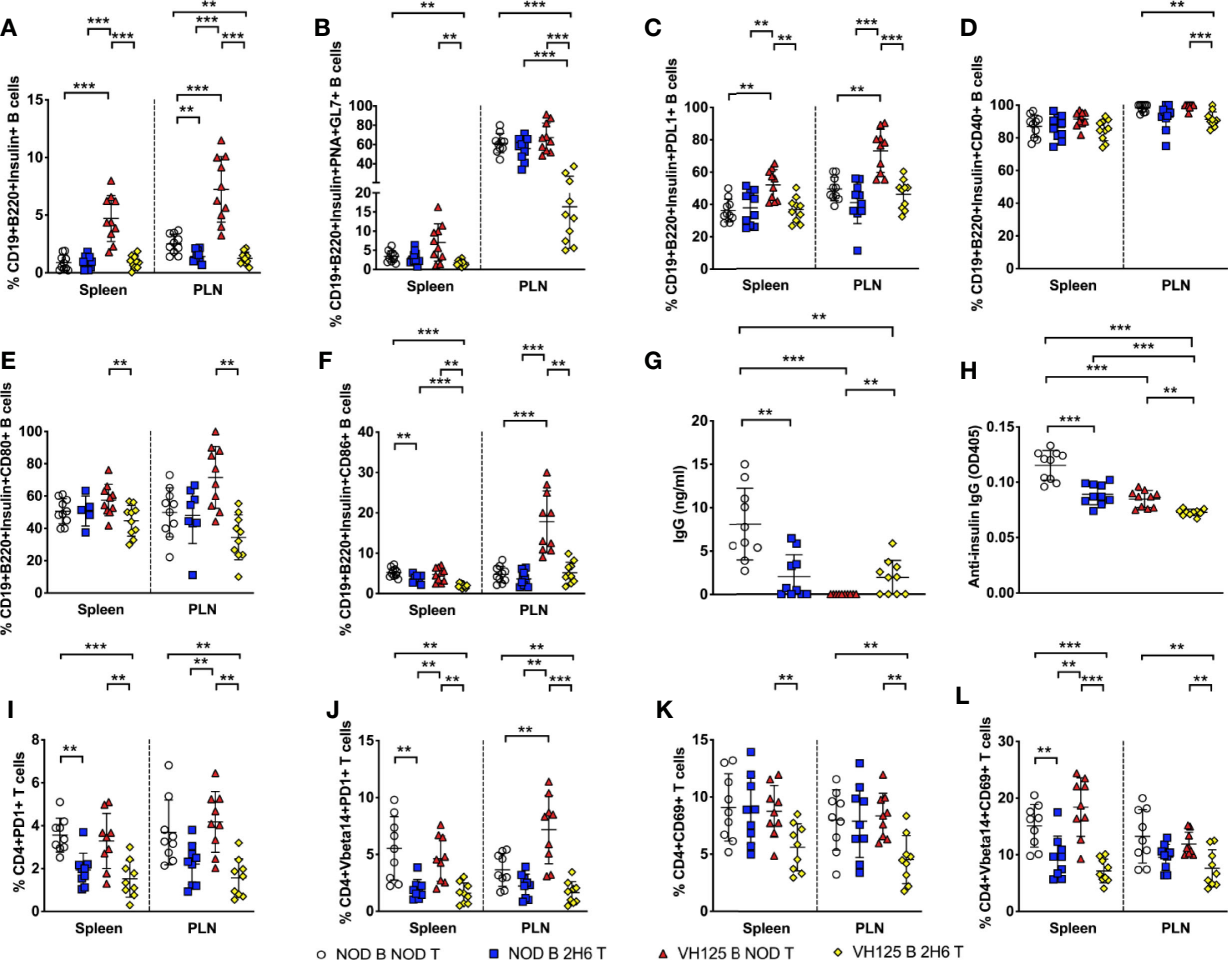
2H6 T cells reduce VH125 B cells interactions in vivo in Rag-deficient NOD mice. 4-week-old Rag-/-NOD mice were adoptively transferred *i.v.* with 3 × 10^6^ CD4+ T cells together with B cells in a 1:1 ratio, using 8-week-old donor NOD, 2H6 NOD and VH125 NOD mice. 4 weeks post-transfer, splenocytes and cells from the pancreas draining lymph nodes (PLN) were harvested from 8-week-old mice and stained using antibodies for flow cytometry. **(A)** % of Insulin-reactive B cells gated from CD19+B220+TCRβ- live single cells. % Insulin-reactive B cells expressing PNA and GL7 **(B)**, PDL1 **(C)**, CD40 **(D)**, CD80 **(E)**, and CD86 **(F)**, gated from **(A)**. Sera from the same mice were used to measure total class-switched IgG **(G)** and total insulin-reactive IgG **(H)** by ELISA. **(I)** % CD4+PD1+ T cells was gated on live single CD19-CD8- cells with the same gating applied to CD4+PD1+TCRVbeta14+ T cells shown in **(J)**. **(K)** % CD4+CD69+ T cells in total CD4+ T cells **(K)** and TCRVbeta14+CD4+ T cells **(L)**. Data were generated from nine individual mice pooled from two independent experiments. Data from left to right: NOD B and NOD T, NOD B and 2H6 T, VH125 B and NOD T, VH125 B, and 2H6 T are shown in this order in each plot. Data from NOD B and NOD T cell recipient Rag-/-NOD mice are shown as a non-transgenic mouse comparison. Data were assessed for significance using multiple T tests and FDR correction. **P < 0.001, ***P < 0.0001.

To confirm our *in vitro* findings in an *in vivo* setting, we co-transferred CD4+ T cells from NOD or 2H6 NOD mice together with B cells isolated from NOD or VH125 NOD mice into Rag-deficient NOD mice. We investigated the above phenotypes in the recipients, 4 weeks post-transfer. We found that the Rag-deficient mice that were infused with VH125 B cells with 2H6 CD4+ T cells had reduced insulin-specific B cells compared to the mice receiving VH125 B cells and NOD CD4+ T cells (**Figure 6A**). In addition, we found that the Rag-deficient recipients transferred with both 2H6 CD4+ T cells and VH125 B cells had reduced insulin-specific GC B cells, particularly in the PLN compared to all other groups (**Figure 6B**). Similar to the *ex-vivo* results shown in **Figure 5**, we found reduced PDL1+ insulin-specific B cells in the Rag-deficient recipients that were co-transferred with 2H6 CD4+ T cells and VH125 B cells compared to the recipients that received NOD CD4+T cells and VH125 B cells (**Figure 6C**). Moreover, the Rag-deficient recipients that were co-transferred with 2H6 CD4+ T cells and VH125 B cells had reduced co-stimulatory molecules on the insulin-specific B cells (**Figures 6D–F**). All of these observations mirror our findings from the *in vitro* experiments. Although the total concentration of IgG was not the lowest (**Figure 6G**), the level of insulin-reactive IgG antibodies was the lowest in the Rag-deficient recipients co-transferred with 2H6 CD4+ T cells and VH125 B cells compared to all the other groups (**Figure 6H**). Again, total IgG2a and IgG2b isotypes were also increased in the recipients of co-transferred 2H6 T and VH125 B cells compared to NOD T and VH125 B cells, while insulin-reactive IgG1 antibodies were lower but insulin-reactive IgG2b were higher between these co-transferred groups (**Supplementary Figure 4**). The presence of IgG in Rag-deficient recipients that received VH125 B cells in the adoptive transfer model system provides further evidence that the 2H6 CD4+ T cells can induce isotype switching in the VH125 B cells (**Figure 6G**).

Unlike the *ex-vivo* results shown in **Figure 5**, we did not find any changes in the proportion or phenotype of the Tfh cells in the *in vivo* experimental setting (data not shown). However, similar to the data presented in **Figure 5**, we observed reduced PD1+ 2H6 CD4+ T cells, both TCRVβ14- and TCRVβ14+ in the recipients infused with 2H6 T cells, regardless of the source of B cells (**Figures 6I–J**). Resembling the *ex-vivo* results shown in **Figure 1**, we demonstrated that co-transfer of 2H6 CD4+ T cells and VH125 B cells led to the reduction of activated CD4+ T cells expressing CD69 compared to the other recipient groups (**Figures 6K, L**). Overall, our results suggest that the 2H6 CD4+ T cells can alter the phenotype and function of VH125 B cells leading to a more tolerogenic environment.

## Discussion

In this study, we report the regulation of pathogenic insulin-reactive VH125 B cells by insulin-reactive 2H6 T cells promoting tolerance, and protecting NOD mice from T1D development. 2H6 T cells mediate this protection by reducing the insulin-reactive B cell populations and their ability to bind insulin. Furthermore, the 2H6 T cells downregulate the expression of MHC and costimulatory molecules on insulin-reactive B cells and alter germinal center interactions. In addition, 2H6 T cells influence insulin antigen, protein and peptide presentation by VH125 B cells from 2H6VH125 mice and result in weaker T cell proliferation compared to the VH125 B cells from VH125 mice, unless a higher dose of antigen is used. Interestingly, the weaker proliferation is associated with greatly enhanced TGFβ secretion. It would be of interest, in experiments in the future, to test whether the changes induced in VH125 B cells result in subsequent suppression of pathogenic insulin-reactive T cells. *In vivo*, most antigen-specific T-B cell interactions take place in germinal centers and germinal center B cells in 2H6VH125 mice have a significantly reduced expression of PDL1, which was mirrored by reduced PD1 expression on the 2H6 T cells in these mice compared to the NOD mice carrying only VH125 BCR or 2H6 TCR, respectively. The PD1-PDL1 axis is important to keep the host’s immune response in check and inhibition of PD1-PDL1 enhances T cell function. The reduction of PD1 and PDL1 expression may explain the suppressive effect of 2H6 T cells on insulin-reactive B cells and the elevation of the immune-regulatory cytokine TGFβ secretion. Interestingly, IgG antibodies were higher in 2H6VH125 NOD mice than in VH125 NOD mice, suggesting that these 2H6 T cells promote class switching in the insulin-reactive B cells, which has not previously been reported. The class switching is also supported by up-regulation of *aicda*, *tgfbr1*, and *il6* gene expression. Taking an adoptive transfer approach, we were able to confirm the findings in Rag-/-NOD hosts. Our results provide evidence that insulin-reactive and TGFβ-secreting 2H6 regulatory T cells play a dominant role in modulating insulin-reactive pathogenic B cells. This change in the insulin-reactive B cells to a more tolerogenic phenotype could be tested in the future by co-transfer with pathogenic insulin-reactive T cells to the Rag-/-NOD hosts.

Germinal centers are pivotal to the generation of an antigen-specific immune response and are specialized sites in peripheral lymphoid tissues where T:B cell interaction take place. IL-21 is an important cytokine for germinal center formation (35), and is required for the development of T1D in NOD mice (36, 37). Interestingly, the overexpression of IL-21 in pancreatic β cells of C57BL/6 mice resulted in islet destruction and the development of spontaneous T1D in a normally diabetes-resistant C57BL/6 mouse strain (37). The significant reduction of circulating IL-21 in the 2H6VH125 NOD mice in our study further supports the role of IL-21 in the immunopathogenesis of T1D development.

In line with the reduction of IL-21, our study also revealed a reduction of the germinal center B cells and reduced ability of germinal center B cell to interact with Tfh cells in the 2H6VH125 NOD mice, due to the presence of the 2H6 T cells. The reduction was likely mediated by the combination of reduced expression of costimulatory molecules on insulin-reactive B cells, reduced insulin binding by VH125 BCR and an altered cytokine environment, namely lower IL-21 and higher TGFβ. Interestingly, IL-21 can also alter costimulation on B cells (38), and thus, the lower IL-21 may have directly resulted in reduced expression of costimulatory molecules in the B cells of 2H6VH125 NOD mice. These changes on B cells can in turn affect T cell function, which include less proliferation in response to the cognate antigen presented by the B cells, as seen in 2H6VH125 NOD mice. Our studies are interesting in the light of findings that patients with T1D have increased IL-21 and Tfh cells in the blood compared to healthy controls, particularly in children who were positive for multiple autoantibodies prior to T1D diagnosis (39, 40). Furthermore, a longitudinal study assessing the transition of naïve CD4+ T cells to activated memory autoreactive T cells from the blood of infants found that increases in IL-21 and Tfh gene signatures were associated with later development of β cell autoimmunity in children (41, 42).

In our study, we found that the insulin-reactive regulatory 2H6 cells reduced the proportion of Tfh cells and altered the germinal center B cells. In comparison, Wan and colleagues recently reported the interaction of insulin-reactive pathogenic CD4 T cells with VH125 B cells, and the authors found increased Tfh cells and germinal center B cells, which enabled the mice to develop T1D (27). Our results together with the results of Wan and colleagues suggest that antigen-specific T:B cell interactions are vital for both the protection from and promotion of T1D development and that the endpoint is determined by the nature of the T cells, i.e., regulatory vs. pathogenic, which predominantly interact with the germinal center B cells. However, our results additionally showed that the B cells will, in turn, affect T cell functions. Interestingly, when the pathogenic insulin-reactive 8F10 TCR transgene and VH125 BCR transgene are both present, the B cells are able to class switch to IgG subtypes of both insulin-specific autoantibodies and non-insulin-specific antibodies (27). However, the authors used a site directed VH125 transgene that allows class switching, whereas the insulin-reactive BCR, VH125, used in our study is an IgM^a^ transgene, which usually prevents class switching. The presence of regulatory 2H6 T cells in our study also promotes IgG class switching in predominantly non-insulin specific antibodies; however, as the VH125 mice we studied were not on a Rag-deficient background, it is likely these class-switched antibodies were potentially due to escaping B cells that failed to express the VH125 BCR (<5% B cells). This may also contribute to T1D prevention, and thus further investigation will be undertaken in the future.

In conclusion, we have demonstrated that a regulatory insulin-specific T cell promotes tolerance in an insulin-specific B cell, by decreasing T:B cell interactions, but enhancing TGFβ secretion. Uniquely, the presence of 2H6 promotes B cells from the VH125 transgenic mouse to undergo class switching to non-insulin specific IgG, a previously unreported phenomenon, and this may also aid in protecting NOD mice from T1D development. Our study also identifies the down regulation of costimulation and antigen presentation function of insulin-reactive B cells by 2H6 T cells, which counter-regulate the insulin-reactive T cells. Our study further illustrates the importance of enhancing antigen-specific T cell regulation, and the knowledge gained in this study may aid better therapeutic strategies in prevention or treatment of T1D.

## Data Availability Statement

The raw data supporting the conclusions of this article will be made available by the authors, without undue reservation.

## Ethics Statement

The animal study was reviewed and approved by Yale University Institutional Animal Care and Use Committee.

## Author Contributions

JP, YyL, and LW designed the experiments. JP, YyL, MM-S, JG, and LZ conducted the experiments. JP, YyL, MM-S, JG, LZ, FSW, and LW analyzed data. FSW and LW supervised the study. JP, FSW, and LW wrote the manuscript. This project was conceived by LW who assumes responsibility for the work. All authors contributed to the article and approved the submitted version.

## Funding

This work was funded by a JDRF Postdoctoral Fellowship (3-PDF-2016-197-A-N) and more recently a MRC Career Development Award (MR/T010525/1) to JP, MRC research grant MR/K021141/1 to FW and by the Molecular & Genetic Mouse Core of NIH P30 (DK 045735), Diabetes Research Connection and NIH RO1HD097808 to LW.

## Conflict of Interest

The authors declare that the research was conducted in the absence of any commercial or financial relationships that could be construed as a potential conflict of interest.

## Supplementary Material

Supplementary Figure 1 2H6 protects 2H6VH125 mice from insulitis and diabetes development. **(A)** Female mice were monitored for glycosuria weekly, from 12 weeks of age, for spontaneous diabetes development, with diabetes confirmed with a blood glucose >250mg/dl. **(B)** Pancreata from 12-week old female NOD, 2H6, VH125 and 2H6VH125 NOD mice were assessed for immune infiltration from 127, 44, 111, 191 islets respectively (n=9, 4, 11, 11 mice respectively). The insulitis was scored by the percentage of infiltration in each observed islet. Data was assessed for significance using individual Chi-square tests **(A)** or Log-rank (Mantel-Cox) test **(B)**. **p < 0.01, ***p < 0.0001.

Supplementary Figure 2 2H6 T cells can alter total B cell populations. Splenocytes (and pancreas draining lymph nodes (PLN)) were harvested from 8-week-old mice, including the heavy and light chain insulin-specific BCR transgenic NOD mouse (125tg) and stained using antibodies for flow cytometry. **(A)** Absolute cell number from the Spleen and PLN. **(B)** Representative flow staining of insulin-reactive B cells identified by binding to insulin-FITC gated from live single CD19+B220+TCRβ- B cells; **(C)** % of Insulin-reactive B cells summarized from B. **(D)** % Follicular B cells gated on CD19+B220+CD21+CD23+ cells from live single TCRβ- cells and **(E)** % marginal zone B cells gated on CD21+CD23- from the same gate as D. **(F)** Mean Fluorescent Intensity (MFI) of MHCII (IAg7) gated within the total B cell population. G-I; % of CD19+B220+ B cells expressing CD40 **(G)**, CD80 **(H)**, and CD86 **(I)** gated as in B. Data were generated from 9-10 individual mice, pooled from 3-5 independent experiments. Data left to right: NOD, 2H6, VH125, 2H6VH125 are shown in this order in each plot. Data from NOD mice are shown as a non-transgenic mouse comparison. Data were assessed for significance using multiple T tests and FDR correction. **P < 0.001, ***P < 0.0001.

Supplementary Figure 3 2H6 TCR transgene promotes IgG class switching in VH125 B cells. Sera from 8-week old mice were tested for IgG1 **(A)**, IgG2a **(B)**, IgG2b **(C)**, IgA **(D)** and insulin-specific autoantibodies of the same isotypes **(E-H)**. Data were generated from 9-10 individual mice, pooled from 2 independent experiments. Data left to right: NOD, 2H6, VH125, 2H6VH125 are shown in this order in each plot. Data from NOD mice are shown as a non-transgenic mouse comparison. Data were assessed for significance using multiple T tests and FDR correction. **P < 0.001, ***P < 0.0001.

Supplementary Figure 4 2H6 T cells promote IgG class switching in VH125 B cells in vivo in Rag-deficient NOD mice. 4-week-old Rag-/-NOD mice were adoptively transferred i.v. with 3x106 CD4+ T cells co-transferred in a 1:1 ratio with B cells using 8-week-old donor NOD, 2H6 NOD and VH125 NOD mice. 4 weeks post-transfer sera were tested for IgG1 **(A)**, IgG2a **(B)**, IgG2b **(C)**, IgA **(D)** and insulin-specific autoantibodies of the same isotypes **(E-H)**. Data were generated from 9-10 individual mice, pooled from 2 independent experiments. Data left to right: NOD, 2H6, VH125, 2H6VH125 are shown in this order in each plot. Data from NOD B and NOD T recipient mice are shown as a non-transgenic mouse comparison. Data were assessed for significance using multiple T tests and FDR correction. **P < 0.001, ***P < 0.0001.
